# Percutaneous coronary intervention versus medical therapy in patients with angina and grey-zone fractional flow reserve values: a randomised clinical trial

**DOI:** 10.1136/heartjnl-2019-316075

**Published:** 2020-02-29

**Authors:** Barry Hennigan, Colin Berry, Damien Collison, David Corcoran, Hany Eteiba, Richard Good, Margaret McEntegart, Stuart Watkins, John D McClure, Kenneth Mangion, Thomas Joseph Ford, Mark C Petrie, Stuart Hood, Paul Rocchiccioli, Aadil Shaukat, Mitchell Lindsay, Keith G Oldroyd

**Affiliations:** 1 Cardiology Department, Golden Jubilee National Hospital, Glasgow, United Kingdom; 2 BHF Glasgow Cardiovascular Research Centre, Institute of Cardiovascular and Medical Science, University of Glasgow, Glasgow, UK; 3 Cardiology Department, The Mater Private Hospital Cork, Cork, Ireland

**Keywords:** fractional flow reserve, combined pressure and doppler flow coronary wire, percutaneous coronary intervention, stress perfusion MRI

## Abstract

**Introduction:**

There is conflicting evidence regarding the benefits of percutaneous coronary intervention (PCI) in patients with grey zone fractional flow reserve (^GZ^FFR artery) values (0.75–0.80). The prevalence of ischaemia is unknown. We wished to define the prevalence of ischaemia in ^GZ^FFR artery and assess whether PCI is superior to optimal medical therapy (OMT) for angina control.

**Methods:**

We enrolled 104 patients with angina with 1:1 randomisation to PCI or OMT. The artery was interrogated with a Doppler flow/pressure wire. Patients underwent Magnetic Resonance Imaging (MRI) with follow-up at 3 and 12 months. The primary outcome was angina status at 3 months using the Seattle Angina Questionnaire (SAQ).

**Results:**

104 patients (age 60±9 years), 79 (76%) males and 79 (76%) Left Anterior Descending (LAD) stenoses were randomised. Coronary physiology and SAQ were similar. Of 98 patients with stress perfusion MRI data, 17 (17%) had abnormal perfusion (≥2 segments with ≥25% ischaemia or ≥1 segment with ≥50% ischaemia) in the target ^GZ^FFR artery. Of 89 patients with invasive physiology data, 26 (28%) had coronary flow velocity reserve <2.0 in the target ^GZ^FFR artery. After 3 months of follow-up, compared with patients treated with OMT only, patients treated by PCI and OMT had greater improvements in SAQ angina frequency (21 (28) vs 10 (23); p=0.026) and quality of life (24 (26) vs 11 (24); p=0.008) though these differences were no longer significant at 12 months.

**Conclusions:**

Non-invasive evidence of major ischaemia is uncommon in patients with ^GZ^FFR artery. Compared with OMT alone, patients randomised to undergo PCI reported improved symptoms after 3 months but these differences were no longer significant after 12 months.

**Trial registration number:**

NCT02425969.

## Introduction

As percutaneous coronary intervention (PCI) has evolved, it has become increasingly important to accurately identify those patients most likely to derive symptomatic benefit. The use of fractional flow reserve (FFR) to guide decision making has been given a class I, level of evidence A indication by the European Society of Cardiology.^1^ The original clinical validation used a combination of treadmill exercise stress testing, myocardial perfusion imaging with thallium and dobutamine stress echo (DSE) determined that the threshold whereby a coronary stenosis was highly likely to be capable of inducing significant myocardial ischaemia was ≤0.75.^2^ Using this cut-off value, a high concordance between FFR and stress perfusion MRI has been confirmed.^3 4^ The clinical utility of this FFR cut-off value was established in the DEFER study in which it was shown that it was safe to defer PCI in lesions with an FFR ≥0.75.^5^ In order to improve sensitivity and minimise the risk of undertreatment, an FFR cut-off value of ≤0.80 was adopted in the FAME and FAME-2 trials^6 7^ both of which demonstrated improved outcomes with FFR guidance.^8^ Consequently, there is an FFR grey-zone (^GZ^FFR) between 0.75 and 0.80 within which the need to perform revascularisation is less clear.^1^ Importantly, a meta-analysis of pooled FFR and outcome data in medically treated and revascularised patients demonstrated that the optimal FFR cut-off value with regard to major adverse cardiac events (MACE) defined as death, MI and revascularisation was <0.75 on a study level analysis and 0.67 on a patient level analysis.^9^

To date, most studies in patients with ^GZ^FFR values have been retrospective, small, non-randomised and limited by selection bias with conflicting outcomes at follow-up.^10–15^ One retrospective observational study by Adjedj *et al* reported no significant difference in MACE for ^GZ^FFR patients treated with PCI plus optimal medical therapy (OMT) or OMT alone after 5 years of follow-up, 11.2% vs 13.9%, p=0.3.^16^ These findings were confirmed in the ^GZ^FFR cohort of the IRIS-FFR Registry with MACE rates of 8.1% in the deferred group vs 8.4% in the PCI group at a median follow-up of 2.9 years, p=0.79.^17^ This randomised trial was designed to assess whether PCI in patients with ^GZ^FFR values would be associated with improvements in symptoms and whether any such improvement could be predicted by preprocedural myocardial perfusion imaging or by invasive physiological indices of stenosis severity other than FFR itself.

## Methods

The ^GZ^FFR study enrolled patients from the West of Scotland Regional Heart and Lung Centre in the Golden Jubilee National Hospital (GJNH), Glasgow, UK. Ethical approval was obtained from the West of Scotland Research Ethics Committee. There was no formal Patient and Public Involvement in the design or conduct of this trial.

### Inclusion and exclusion criteria

**Figure 1:**
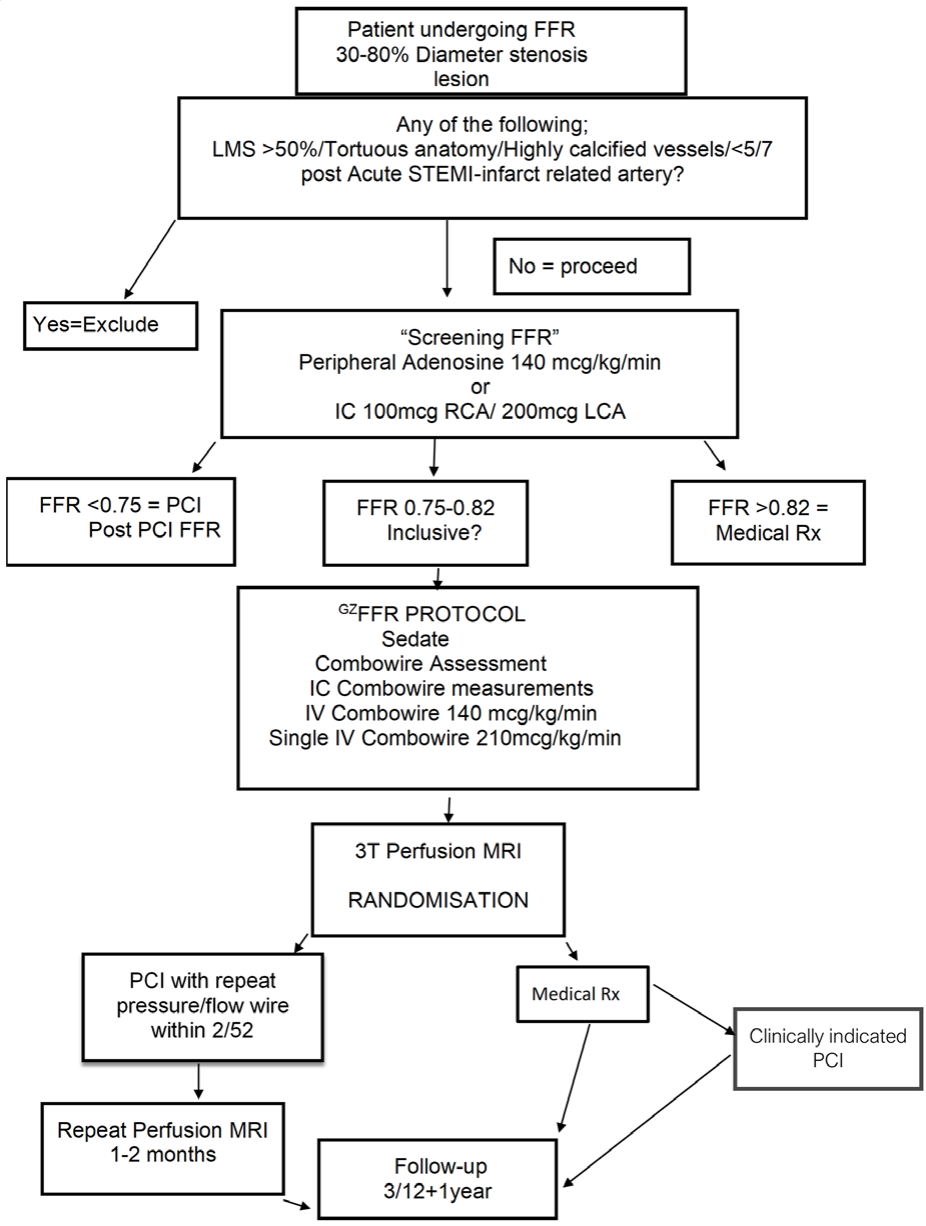
^GZ^FFR flowchart. ‘Screening FFR’ used any pressure wire system for basic FFR assessment without flow indices. All subsequent measurements involved the Combowire device to assess indices of pressure, flow and resistance. FFR, fractional flow reserve; ^Gz^FFR, grey zone; OMT, optimal medical therapy; PCI, percutaneous coronary intervention and optimal medical therapy group; Pd/Pa, resting pressure gradient; QCA, quantitative coronary angiography; STEMI, ST elevation myocardial infarction.

We recruited consecutive consenting patients with a clinical indication for a pressure wire based diagnostic evaluation of an intermediate coronary lesion (30%–80% diameter stenosis by visual assessment) who had ^GZ^FFR values between 0.75 and 0.82. Patients were eligible if they had either stable angina or residual non-culprit disease after non-ST elevation myocardial infarction or ST elevation myocardial infarction (STEMI) following treatment of the culprit vessel. In patients returning for planned pressure wire studies following culprit vessel PCI, a minimum interval of 2 weeks from the index event was required and symptoms had to be stable for ≥3 days. Patients with excessive tortuosity, calcification or left main disease with ≥50% diameter stenosis were excluded (figure 1). Patients were ineligible for randomisation in the event of residual untreated obstructive coronary disease in another vessel ≥2 mm in calibre.

### Design

Between May 2015 and October 2016, we recruited 104 patients who were randomised 1:1 to PCI with OMT (PCI) or OMT alone using sealed envelopes provided by the Robertson Centre for Biostatistics, University of Glasgow. They were stratified by gender, diabetic status and 65 years of age cut-off. The primary outcome measure was angina status assessed using the Seattle Angina Questionnaire (SAQ). Patients completed their first SAQ immediately following baseline coronary angiography and invasive physiology studies except for patients who underwent PCI of another non ^GZ^FFR vessel at the time of recruitment where the SAQ was completed at least 4 weeks after the initial PCI. At 3 months following randomisation, the second SAQ was administered over the telephone by a research nurse who was blinded to treatment group and all other data. Recruitment was stopped when 104 patients had undergone successful randomisation following discussion with the trial biostatistician as our loss to follow-up rate was lower than expected.

### Non-invasive ischaemia assessment

This was performed using stress perfusion MRI. Standard techniques were employed with three short axis cuts during rest and adenosine stress phases and administration of a bolus of 0.05 mmol/kg gadolinium. Study participants, clinicians and clinical research nurses were all blinded to the perfusion MRI. All images were analysed on a Medis Suite 2.1.12.2 Medis Medical Imaging Systems (Leiden, The Netherlands) workstation by two cardiologists with Level 3 accreditation with the European Association of Cardiovascular Imaging (CB and DC). Both datasets were then adjudicated in the event of discordance a third observer (SW). Images were assessed according to 16 segment American Heart Association analysis to define the extent of ischaemia. The definition of significant reversible ischaemia was ≥2 segments with ≥25% ischaemia or ≥1 segment with ≥50% ischaemia similar to recent studies.^18^ Patients unable to undergo MRI studies were assessed using DSE.

### Medical therapy and titration of antianginal drugs

All of the participants received OMT in line with contemporary guidelines with titration of antianginals as clinically appropriate^19^ though this was patient driven and not performed according to a preset protocol. Patients with persistent or recurrent angina postrandomisation underwent titration of medications supervised by the clinical research staff. When a treatment change was indicated by ongoing anginal symptoms, the patient was contacted in person or by telephone followed up by email or letter and advised to attend their GP in order to have their prescription modified accordingly. All patients were directed to follow-up with the research team within 2 weeks to assess the clinical response and to notify the research team in the event of either a failure to control angina, hospitalisation or other clinical event. Beyond that, patients had email and phone access to a consultant cardiologist for purposes of management of poorly controlled symptoms. Patients could cross over from the OMT group to the PCI group at any time during follow-up though they were encouraged to persist with the assigned treatment group for 3 months if feasible.

### Coronary physiology studies

Following identification of a vessel with a ^GZ^FFR value using a conventional pressure wire, a Phillips Volcano Combowire was equalised and introduced into the distal third of the target coronary artery. An intracoronary bolus of 200 µg of isosorbide dinitrate was administered followed by intravenous infusion of adenosine at 140 mcg/kg/min. After data acquisition, the wire was withdrawn to check for pressure drift. Drift ≥0.03 was considered unacceptable necessitating repeated measurements. All data were anonymised and analysed with Combomap V.1.9 software. Each recording was reviewed with attention to flow signal quality and pressure signals for pressure damping with classification of results according to published cut-offs.^20^

### Coronary angiography and PCI

Coronary angiography was performed as per standard practice in the in Golden Jubilee National Hospital (GE Innova 2121 and 2100). Quantitative coronary analysis was performed on a workstation using computer-assisted planimetry QAngio XA 3D 1.0 (MEDIS, Leiden). An APPROACH Score was calculated for each lesion in order to evaluate the volume of myocardium subtended by the vessel of interest.^21^ PCI was performed according to international guidelines.^1^

### Statistics and sample size calculation

The study design and final analysis involved an experienced biostatistician (JDM). We required 108 subjects to provide 90% power at a multiple testing adjusted 5% level of significance to detect a clinically relevant difference of 10 points for each of the five components of the SAQ between the OMT and PCI groups assuming a within group SD of 18 points as per similar studies.^22^ We expected loss to follow-up of 10%. T-tests or χ² tests were used where appropriate. A posthoc analysis using ANCOVA was performed using Hochberg’s FWER procedure. We explored the ability of coronary flow velocity reserve (CFVR), Hyperaemic Stenosis Resistance Index (HSR) and hyperaemic microvascular resistance (HMR) to predict the presence of ischaemia on stress perfusion MRI using ROC curves using SPSS statistics package V.21.0. Armonk, New York, USA: IBM.

### Results

**Figure 2:**
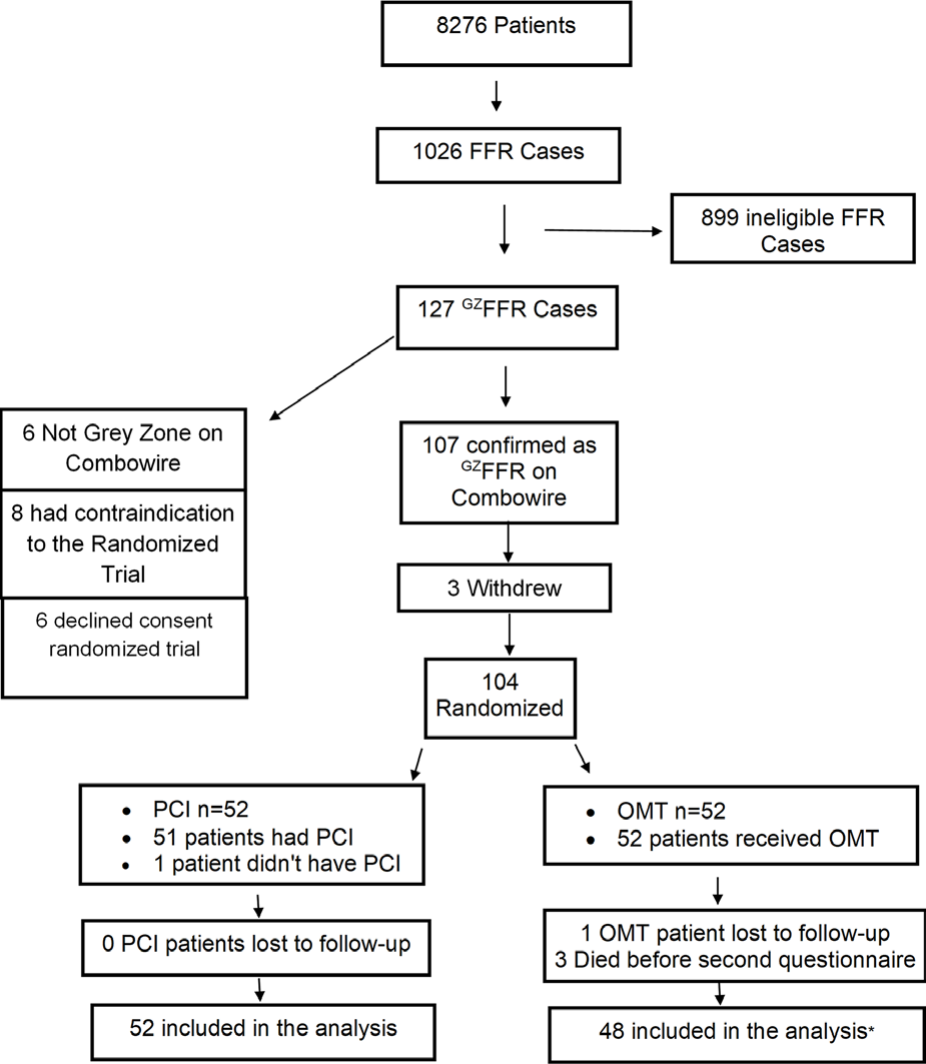
Consort flow diagram for the ^GZ^FFR trial. *One patient died at 65 days postrandomisation following a witnessed fall with traumatic intracranial haemorrhage, another died at 51 days postrandomisation from metastatic lung cancer diagnosed during the MRI performed as part of the study and the third died of pulmonary emboli post resection of a chronic benign meningioma at 84 days postrandomisation. Combowire, combined pressure and Doppler flow wire; FFR, fractional flow reserve; ^GZ^FFR, grey zone; OMT, optimal medical therapy; PCI, percutaneous coronary intervention and optimal medical therapy group.

During the period of this study, we performed FFR assessment in 1026 patients of whom 127 (12.4%) had values of 0.75–0.82 inclusive. All of these patients were invited to participate in the trial and 108 agreed. Subsequently, three patients withdrew consent and one patient had non-^GZ^FFR physiology at the time of planned PCI and was excluded leaving 104 randomised patients (table 1). See table 2 for details regarding clinical presentation. One patient randomised to PCI had severe myocardial bridging adjacent to the target stenosis potentially compromising the safe performance of PCI and was managed medically. One patient randomised to PCI travelled abroad with a resultant delay in the patient receiving their assigned treatment. This patient sustained a spontaneous STEMI in the territory of the target artery at 40 days postrandomisation and underwent emergency PCI. All other patients received their allocated treatment (figure 2) and all results are reported by intention to treat. The mean (SD) number of antianginal drugs in each group at 3 months follow-up was 1.5 (0.7) with OMT only vs 1.3 (0.8) with PCI plus OMT; p=0.15 (online supplementary eTable 1). Drug eluting stents were used in all patients.

10.1136/heartjnl-2019-316075.supp1 Supplementary data

**Table 1:** Risk factors according to treatment strategy and symptom status, previous cardiac history and mode of presentation at time of recruitment

Variable	OMT n=52	PCI n=52
Age	61 (SD 9.0)	60 (SD 8.0)
Male	39 (75%)	40 (76.9%)
Female	13 (25%)	12 (23.1%)
Current smoker	13 (25%)	21 (40.3%)
Previous smoking	13 (25%)	11 (21.1%)
**HTN**	**44 (84.6%)**	**31 (59.6%)**
Hyperlipidaemia	31 (59.6%)	38 (73.1%)
T2DM	10 (19.2%)	10 (19.2%)
IDDM	2 (3.8%)	2 (3.8%)
FHX CAD	38 (73.1%)	33 (63.5%)
PVD	4 (7.7%)	6 (11.5%)
Cerebrovascular disease	4 (7.7%)	4 (7.7%)

Significant differences indicated in bold, p value for HTN=0.004.

FHX CAD, family history coronary artery disease; HTN, hypertension; IDDM, insulin dependent diabetes; OMT, optimal medical therapy group; PCI, percutaneous coronary intervention group; PVD, peripheral vascular disease; T2DM, type 2 diabetes mellitus.

**Table 2:** Table 1 Clinical features according to treatment strategy

Variable	OMT (n=52)	PCI (n=52)
NYHA Class		
1	31 (59.6%)	39 (75%)
2	13 (25%)	9 (17.3%)
3	4 (7.6%)	2 (3.8%)
4	3 (5.7%)	2 (3.8%)
CCS Class*		
1	11 (21.2%)	14 (26.9%)
2	30 (57.7%)	30 (57.7%)
3	4 (7.7%)	4 (7.7%)
4	7 (13.5%)	4 (7.7%)
Previous PCI	28 (53.8%)	36 (69.2%)
Previous MI	21 (40.4%)	31 (59.6%)
Presentation		
Stable angina	32 (61.5%)	21 (40.4%)
Non-culprit NSTEMI	12 (23.1%)	17 (32.7%)
Unstable angina	3 (5.8%)	3 (5.8%)
Non-culprit STEMI	5 (9.6%)	11 (21.2%)

CCS Class may not be indicative of anginal class as per Seattle Angina Questionnaire in the setting of non-culprit disease where scores were calculated at a minimum of 4 weeks post initial PCI in order to ensure scores were reflective of angina from the ^GZ^FFR vessel under study.

CCS, Canadian Cardiovascular Society; NSTEMI, non-ST elevation myocardial infarction; OMT, optimal medical therapy group; PCI, percutaneous coronary intervention; STEMI, ST elevation myocardial infarction.

#### Baseline quantitative coronary angiography

The coronary arteries with ^GZ^FFR values were the left anterior descending in 79 patients (76%), the right in 14 patients (14%) and the left circumflex in 8 patients (8%). See online supplementary eTable 2 for further segmental breakdown. There was no significant difference between groups in terms of any baseline QCA variables (table 3).

**Table 3:** Quantitative coronary angiographic data according to treatment strategy

Variable	OMT (n=52)	PCI (n=52)
Diameter stenosis (%)	44 (8)	45 (10)
Area stenosis (%)	69 (8)	69 (10)
Lesion length (mm)	10 (4)	10 (4)
APPROACH Score (%)	32 (9)	32 (8)

OMT, optimal medical therapy group; PCI, percutaneous coronary intervention group.

##### Invasive coronary physiology

In addition to the qualifying FFR measurement, 89/93 patients had additional measurements of CFVR that were of sufficient quality for analysis. There were no significant differences between the groups at baseline with mean (SD) FFR value of 0.78 (0.02). While all patients had ^GZ^FFR physiology confirmed on their first pressure wire assessment at enrolment, before Doppler flow interrogation, there was less Doppler flow data available in the OMT versus PCI group (n=38 vs 51, respectively) as there was a second opportunity to acquire flow data in patients reattending for PCI with protocol mandated repeat physiology pre-PCI. Potentially ischaemic values of CFVR (<2.0) and HSR (≥0.8 mm Hg/cm/s) were observed in 26/89 (29%) and 7/89 (8%) of patients, respectively. HMR was elevated (>2.3 mm Hg/cm/s) in 27/89 (30%) of cases (online supplementary eFigure 1). A total of 42/52 (78.9%) of patients undergoing PCI had both pre-PCI and post-PCI invasive physiology assessment. FFR pre-PCI was 0.77 (0.02) compared with 0.90 (0.06) post-PCI; mean delta 0.12 (95%CI 0.11 to 0.15), p=0.0001 (table 4).

**Table 4:** Physiology for all patients with Combowire data including post-PCI Combowire results pressure following Core Laboratory analysis (n=89/93), flow and resistance data for all randomised patients with Combowire data according to treatment group

Baseline invasive physiology for entire cohort				
	N	Minimum	Maximum	Mean (SD)
FFR	89	0.75	0.82	0.78 (0.02)
HMR	89	0.9	6.9	2.10 (0.84)
HSR	89	0.15	2.00	0.52 (0.25)
CFVR	89	1.3	5.0	2.41 (0.75)

By randomised group				
		N	Mean	SD
FFR	OMT	38	0.78	0.02
	PCI	51	0.78	0.02
HMR	OMT	38	2.21	0.74
	PCI	51	2.01	0.91
HSR	OMT	38	0.55	0.21
	PCI	51	0.50	0.27
CFVR	OMT	38	2.35	0.72
	PCI	51	2.45	0.77

Invasive physiology pre-PCI and post-PCI				
	Pre-PCI	Post-PCI	N	P value
FFR	0.77 (0.02)	0.90 (0.06)	41	0.001
HMR	2.07 (0.98)	2.31 (0.95)	41	0.02
HSR	0.51 (0.29)	0.20 (0.18)	40	0.001
CFVR	2.34 (0.73)	2.28 (0.69)	40	0.548

CFVR, coronary flow velocity reserve; FFR, fractional flow reserve; HMR, hyperaemic microvascular resistance; HSR, Hyperaemic Stenosis Resistance Index; N, total number of patients; OMT, optimal medical therapy group; PCI, percutaneous coronary intervention with medical therapy group.

##### Primary outcome measure (SAQ scores)

Of the 104 randomised patients, 100 pairs of SAQ data were available (96% of the randomised cohort) and three patients in the OMT only group died due to non-cardiac causes before the primary endpoint assessment at 3 months (figure 2). A fourth patient, also in the OMT only group, was lost to follow-up but was confirmed to be alive and free of angina 1 year postrandomisation via their family doctor. The within group mean change in the SAQ domain of angina frequency from baseline to 3 months was 10 (23) with OMT alone vs 21 (28) with PCI plus OMT; p=0.04. The within group mean change in the SAQ domain of quality of life (QOL) from baseline to 3 months was 11 (24) with OMT alone vs 24 (26) with PCI plus OMT; p=0.01. There were no significant differences in the SAQ domains of treatment satisfaction, physical limitation or angina stability (table 5 and online supplementary eFigure 2). The within group mean change for the SAQ summary score was 17 (18) with OMT alone vs 25 (21) with PCI plus OMT, p=0.04. Posthoc analysis of covariance (ANCOVA) incorporating baseline SAQ scores and using Hochberg’s FWER error rate adjusted p values confirmed a statistically significant difference between OMT and PCI plus OMT for Anginal Frequency (p=0.04) and QOL (p=0.02). At baseline, there was no between group difference for ‘Freedom from Angina’ (Angina Frequency score=100) but a significant difference between groups was observed at 3 months: 20/48 (41.7%) with OMT alone vs 34/52 (65.4%) with PCI plus OMT, p=0.02.

**Table 5:** Mean change in SAQ scores from baseline to 3 months

SAQ parameter	Group	N	Mean	SD	95% CI of the difference	P value
Summary Score	OMT	48	17	18	+1.5 to +18	0.04
	PCI	52	25	21		
Physical limitation	OMT	48	11	23	−4 to +15	0.28
	PCI	52	16	26		
Anginal stability	OMT	48	−3	34	−14 to +12	0.91
	PCI	52	−3	33		
Anginal frequency	OMT	48	10	24	+1 to +21	0.04
	PCI	52	21	28		
Treatment satisfaction	OMT	48	−4	20	−1 to 12	0.10
	PCI	52	2	13		
Quality of life	OMT	48	11	24	+3 to +23	0.01
	PCI	52	24	26		

A higher value indicates improved clinical status (see online supplementary eTable 3 for baseline values).

OMT, optimal medical therapy group; PCI, percutaneous coronary intervention with medical therapy group; SAQ, Seattle Angina Questionnaire score.

### Non-invasive evidence of ischaemia

A total of 98 patients had perfusion MRI data (online supplementary eTable 4). Of these, 74 (76%) had no detectable ischaemia in the territory of the ^GZ^FFR vessel (online supplementary eTable 5). Five patients were unable to undergo perfusion MRI and had DSE instead. None of this group had detectable ischaemia in the territory of the ^GZ^FFR vessel. There was no significant between group difference in the incidence of ischaemia on non-invasive testing at baseline (p=0.41) (online supplementary eTable 6). Postprocedural MRI data were available in 41/51 patients who were randomised to undergo PCI. Of these, 3/41 (7.3%) still had detectable ischaemia in the ^GZ^FFR territory. Diagnostic accuracy of CFVR, HSR and HMR for predicting major ischaemia on MRI pre randomisation was assessed. HSR was the most predictive at 0.609, (95% CI 0.442 to 0.783) (online supplementary eFigure 3).

### Secondary outcomes at 3 months

This study was not powered to detect a difference in hard clinical endpoints. All-cause mortality at 3 months was 3/52 (5.7%) in OMT group (all confirmed non-cardiac deaths) vs 0/52 (0%) in PCI group.

### 12-month follow-up

After 12 months, 89/100 patients completed another SAQ (online supplementary eTable 7). The within group mean change in the SAQ domain scores from baseline to 12 months remained numerically higher in patients treated by PCI plus OMT versus OMT alone but these differences were no longer statistically significant, possibly due to a loss of power and/or restenosis.

## Discussion

**Figure 3:**
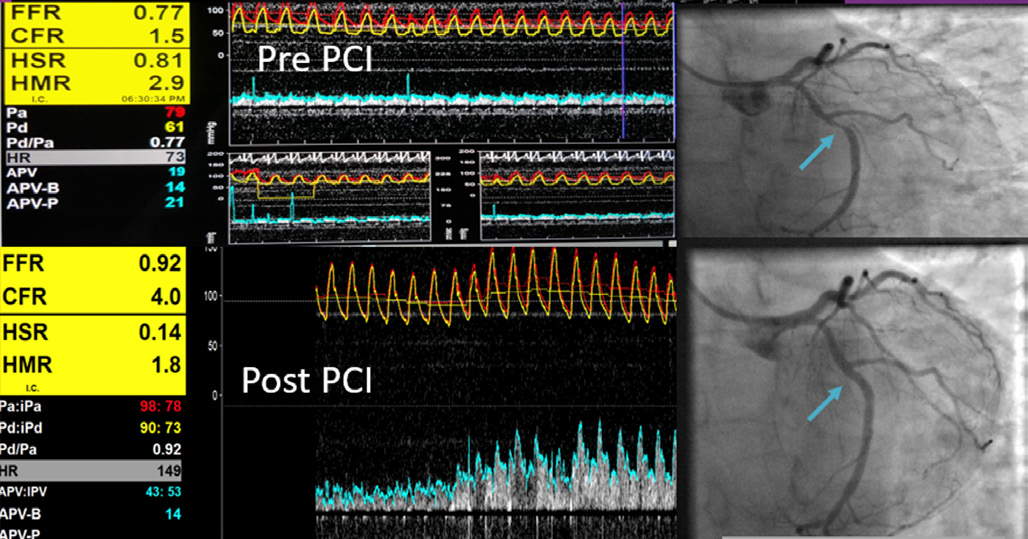
This patient had a moderately severe mid left circumflex lesion with ^GZ^FFR physiology with reduced CFVR of 1.5 pre-PCI which improved to a CFVR of 4 post-PCI. The stenosis resistance HSR reduced following PCI with improved FFR. ^GZ^FFR coronary lesion in mid circumflex indicated by blue arrow before PCI (upper panel) and after PCI with coronary physiology data in the left panel. CFVR, coronary flow velocity reserve; FFR, fractional flow reserve; ^GZ^FFR, grey zone; HSR, Hyperaemic Stenosis Resistance Index.

Prior studies do not indicate that hard clinical events are likely to be reduced by PCI in patients with ^GZ^FFR coronary physiology.^16 17^ As such, most of the justification for PCI in this group of patients must be based on an anticipated improvement in symptoms and quality of life. In this single-centre prospective randomised trial of OMT versus PCI plus OMT in patients with angina and ^GZ^FFR values, patients assigned to PCI reported a significant improvement in symptoms and quality of life as assessed by the SAQ. Freedom from angina was also significantly more frequent in the PCI group. Non-invasive evidence of ischaemia on stress perfusion MRI was only identified in around 1/4 of these patients with ^GZ^FFR although full quantitative perfusion analysis was not performed. Combined pressure and Doppler flow wire technology in its current form is difficult to use but the concept of using flow, pressure and resistance to determine the potential benefit of revascularisation remains attractive (figure 3).^23^ Indeed, HSR was the best invasive physiological index for predicting the presence of perfusion abnormalities with the relatively modest agreement most probably due to the low prevalence of ischaemia in this cohort (online supplementary eFigure 3).

Prior to this study, we had anticipated that a large proportion of patients with angina, coronary disease and ^GZ^FFR physiology considered for treatment with PCI could achieve equivalent symptoms control with medical management only. However, as a group, the patients randomised to PCI demonstrated superior symptom control and quality of life after 3 months and this treatment effect was still apparent after 12 months follow-up. Longer follow-up in a larger scale study will be important to further clarify the roles of medical management and PCI in this group.

### Limitations

The major limitation of this study is that we did not employ a placebo group and, as the patients in this study were aware of their allocated treatment, we cannot out rule a placebo effect. Reassuringly, the magnitude of the improvement in SAQ scores with PCI exceeded that reported in the placebo arm of the ORBITA trial despite the fact that significantly less medical therapy was administered to our PCI group than in ORBITA (1.3 vs 2.9 anti-anginal drugs; online supplementary eTable 8). In our study, 94% of patients had an FFR ≤0.80 compared with only 71% in ORBITA and this may explain the greater improvement observed with PCI.^24^

## Conclusion

We have performed a prospective randomised controlled trial of open-label PCI plus medical therapy versus medical therapy only in patients with angina, coronary disease and ^GZ^FFR values. Despite a relatively low incidence of ischaemia on non-invasive testing, patients treated by PCI reported fewer symptoms and improved quality of life, some of which may have been due to a placebo effect.

**Key questions**

**What is already known on this subject?**

- In the DEFER trial, the safety of deferring percutaneous coronary intervention (PCI) based on fractional flow reserve (FFR) was established with a FFR cut-off value of >0.75. In the FAME trials, improved clinical outcomes were demonstrated if PCI was performed in all lesions with FFR≤0.80. Previous studies suggest that 20%–25% of patients undergoing FFR assessment have values that fall within the grey zone of 0.75–0.80. There is conflicting evidence regarding the benefits of PCI in these patients and no data from randomised clinical trials.

**What might this study add?**

- In patients with grey-zone FFR values randomised to either medical therapy or PCI plus medical therapy, those treated by PCI had less angina and improved quality of life. Some of this benefit could be due to placebo as this trial had no sham control.
- Only 17% of these patients had evidence of significant ischaemia based on stress myocardial perfusion studies using MRI.
- Only 8% of these patients had evidence of significant ischaemia based on hyperaemic stenosis resistance, an invasive physiological index derived from both pressure and Doppler flow measurements.

**How might this impact on clinical practice?**

- Clinicians considering PCI in stenoses with grey-zone FFR values should understand that there is a low incidence of significant myocardial ischaemia in this group. Nevertheless, patients can be advised that there is a reasonable probability of symptomatic improvement, albeit that some of this could be a placebo effect.
