# Supplementary material for: Percutaneous coronary intervention versus medical therapy in patients with angina and grey-zone fractional flow reserve values: a randomised clinical trial
Source: Heart. 2020 Feb 29;106(10):758–64. doi: 10.1136/heartjnl-2019-316075 (PMC7229900; doi:10.1136/heartjnl-2019-316075)
Supplement: Supplementary data [file heartjnl-2019-316075supp001.pdf]

eTable 1. Anti-anginal therapy at 3 months follow-up according to randomized strategy.

Medication	OMT n=48	PCI n=52	P Value
Nitrate	16 (33.3%)	7 (13.5%)	0.018
Ivabradine	0 (0%)	1(1.9%)	0.334
Beta Blocker	41 (85.4%)	40 (76.9%)	0.279
Calcium channel blocker	14 (29.2%)	16 (30.8%)	0.861
Nicorandil	3 (6.3%)	4 (7.7%)	0.778

OMT= Optimal medical therapy, PCI= Percutaneous coronary intervention and optimal medical therapy group. A p value of <0.05 was considered statistically significant.

eTable 2: Breakdown of cases by ^{GZ}FFR vessel.

Grey Zone Vessel	Frequency	% (n=104)
Intermediate	1	1.0
LAD	79	76.0
LCX	8	7.7
LPDA	1	1.0
OM1	1	1.0
RCA	14	13.5
Total	104	100.0

OM1=Obtuse Marginal 1, LCX=Left Circumflex, LPDA= Left Posterior Descending Artery.

eTable 3: *Baseline SAQ scores pre randomisation*

SAQ parameter	Group	N	Mean	SD	P value
Physical limitation	OMT	48	67	21	0.40
	PCI	52	63	28	
Anginal stability	OMT	48	56	27	0.71
	PCI	52	58	26	
Anginal frequency	OMT	48	70	24	0.67
	PCI	52	68	30	
Treatment satisfaction	OMT	48	88	17	0.80
	PCI	52	89	14	
Quality-of-life	OMT	48	54	23	0.71
	PCI	52	52	29	

eTable 4 Baseline MRI Results: Mass and Function

MRI Mass and Function by Treatment Group					
	Group	N	Mean	SD	p
End Diastolic Volume	OMT	50	153.6	36.4	0.637
	PCI	47	156.8	30.9	
End Systolic Volume	OMT	50	64.2	26.6	0.613
	PCI	47	66.6	19.5	
Stroke Volume	OMT	50	88.1	23.3	0.586
	PCI	47	90.4	17.2	
Ejection Fraction	OMT	50	58.9	9.7	0.608
	PCI	47	58.1	6.4	

Entire Cohort	N	Mean	SD
End Diastolic Volume	97	155.2	33.7
End Systolic Volume	97	65.4	23.3
Stroke Volume	97	89.2	20.5
Ejection Fraction	97	58.6	8.3

eTable 5: This table includes all patients with MRI data at enrollment and demonstrates the numbers of patients according to the numbers of segments with detectable ischemia in the ^{GZ}FFR territory.

Total number Grey Zone FFR segments per patient with any detectable ischemia	0	1	2	3	4
Total Patient number (n=98)(%)	74(75.5%)	8(8.2%)	10(10.2%)	5(5.1%)	1(1%)

Note transmurality of ischemia not shown.

eTable 6*:* Major segmental ischemia on MRI according to treatment group.

Group	1 Segment ischemia	2 Segment ischemia	3 Segment ischemia	4 Segment ischemia	Total number of segments with significant ischemia
OMT	4 (7.8%)	8 (15.7%)	2 (3.9%)	1 (2%)	11 (21.6%)
PCI	4 (8.5%)	2 (4.3%)	3 (6.4%)	0 (0%)	6 (12.8%)

This table illustrates the numbers of patients with segmental ischaemia in their ^{GZ}FFR territory according to the total number of segments with ischaemia between 25%-100% transmurality as well as those patients that met the study definition for significant ischemia with (≥2 segments with ≥25% ischemia or ≥1 segment with ≥50% ischemia. OMT= Optimal medical therapy, PCI= Percutaneous coronary intervention and optimal medical therapy group.

eTable 7: 12-month Seattle Angina Score Delta values according to treatment strategy

Questionnaire Parameter	Group	N	Mean delta	Standard. Deviation	P value
SAQ Summary Delta Score	OMT	45	9.8	18	0.208
	PCI	44	15.1	21	
Physical limitation Delta Score	OMT	45	2.9	20	0.07
	PCI	44	11.6	24	
Anginal stability Delta Score	OMT	45	.5	32	0.62
	PCI	44	-2.8	32	
Anginal frequency Delta Score	OMT	45	13.5	25	0.77
	PCI	44	15.2	29	
Treatment satisfaction Delta Score	OMT	45	-2.3	16	0.35
	PCI	44	.9	17	
Quality of Life Delta Score	OMT	45	12.9	24	0.27
	PCI	44	18.5	24	

OMT= Optimal medical therapy, PCI= Percutaneous coronary intervention and optimal medical therapy group. A p value of <0.05 was considered statistically significant. SAQ Summary Score is the average of the sum of the Physical Limitation, Anginal Frequency and Quality of life scores.

eTable 8. This table illustrates the difference in Seattle Angina questionaires in the ORBITA placebo group at 6 weeks versus the ^{GZ}FFR PCI group at 3 months.

Study	SAQ Parameter	SAQ DELTA at Follow-up Mean (SD)	P
^{GZ}FFR PCI	PHYSICAL LIMITATION	16.1 (26.1)	0.008
ORBITA PLACEBO		5 (21·2)	
^{GZ}FFR PCI	ANGINAL FREQUENCY	20.6 (27.6)	0.03
ORBITA PLACEBO		9.6 (28.4)	
^{GZ}FFR PCI	ANGINAL STABILITY	-3.4 (32.9)	0.77
ORBITA PLACEBO		-5.1 (31.6)	

Higher delta scores indicate greater clinical improvement. Note only 3 SAQ parameters for ORBITA were published previously (25), $p<0.05$ is considered significant.

eFigure 1: This figure illustrates a typical ^{GZ}FFR patient.

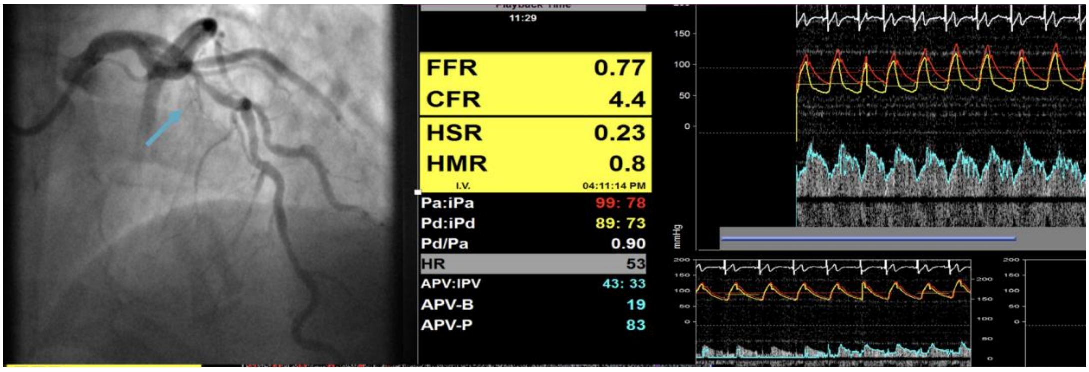

This patient was randomised to medical therapy following baseline angiography (which an intermediate lesion in the proximal LAD with preserved coronary flow reserve and low HSR.

eFigure 2. The Primary outcome: Mean delta in Seattle angina scores according to treatment group for each of the 5 sub- scores of the Seattle angina questionnaire.

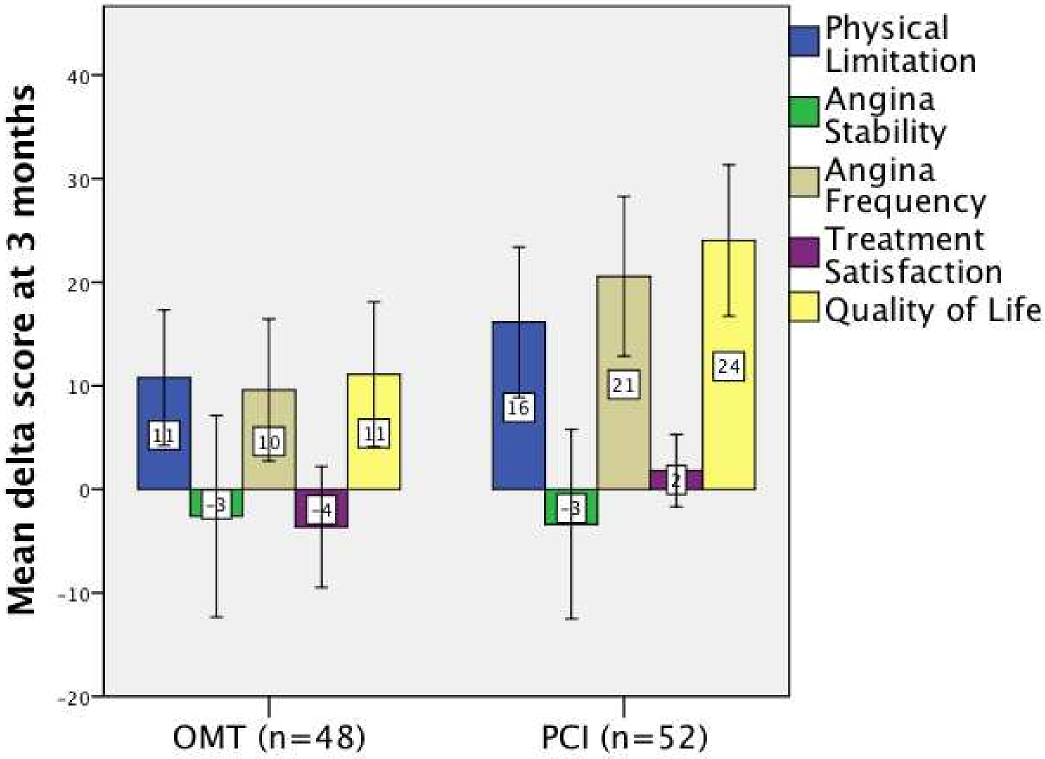

eFigure 3: Receiver Operator Characteristics curve illustrating the diagnostic accuracy of all 4 coronary physiological measurements in reference to major ischaemia on cardiac MRI

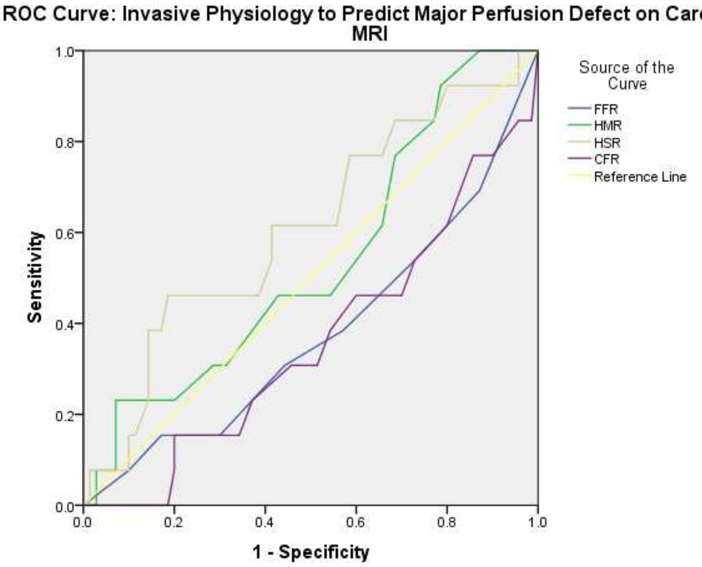

Test	Area Under the Curve	95% Confidence Interval	
		Lower	Upper
FFR	0.379	0.208	0.550
HMR	0.536	0.368	0.703
HSR	0.613	0.442	0.783
CFR	0.362	0.198	0.526
